# Supplementary material for: The Consortium for Genomic Diversity, Ancestry, and Health in Colombia (CÓDIGO): building local capacity in genomics, bioinformatics, and precision medicine
Source: bioRxiv. 2025 May 1:2025.04.28.651081. Preprint. [Version 1] doi: 10.1101/2025.04.28.651081 (PMC12190313; doi:10.1101/2025.04.28.651081)

**The Consortium for Genomic Diversity, Ancestry, and Health in Colombia (CÓDIGO): building local capacity in genomics, bioinformatics, and precision medicine**

Leonardo Mariño-Ramírez^1^, Shivam Sharma^2,3^, James Matthew Hamilton^2^, Thanh Long Nguyen^2^, Sonali Gupta^1,3^, Aravinth Venkatesh Natarajan^2^, Shashwat Deepali Nagar^2^, Jay Landon Menuey^2^, Wei-An Chen^2^, Adalberto Sánchez-Gómez^4^, José María Satizábal-Soto^4^, Beatriz Martínez^5^, Javier Marrugo^5^, Miguel A. Medina-Rivas^6^, Juan Esteban Gallo^1,3^, I. King Jordan^2,3^, Augusto Valderrama-Aguirre^7^

^1^ National Institute on Minority Health and Health Disparities, National Institutes of Health, Bethesda, Maryland, USA

^2^ School of Biological Sciences, Georgia Institute of Technology, Atlanta, Georgia, USA

^3^ IHRC-Georgia Tech Applied Bioinformatics Laboratory, Atlanta, Georgia, USA

^4^ Physiology Sciences Department, School of Health, Universidad del Valle, Cali, Colombia

^5^ Molecular Genetics Lab., Institute for Immunological Research, University of Cartagena, Cartagena, Colombia

^6^ Centro de Investigación en Biodiversidad y Hábitat, Universidad Tecnológica del Chocó, Quibdó, Chocó, Colombia

^7^Department of Biological Sciences, Universidad de Los Andes, Bogotá DC, Colombia

**Contents**

[Supplementary Figure 1. **Variant merging and harmonization** 2](#_Toc173411523)

[Supplementary Table 1. **CÓDIGO datasets** 3](#_Toc173411524)

[Supplementary Table 2. **Global reference populations** 4](#_Toc173411525)

[Supplementary Figure 2. **CÓDIGO development stack** 5](#_Toc173411526)

[Supplementary Figure 3. **K-means clustering elbow plot** 6](#_Toc173411527)

Supplementary Figure 1. **Variant merging and harmonization.**


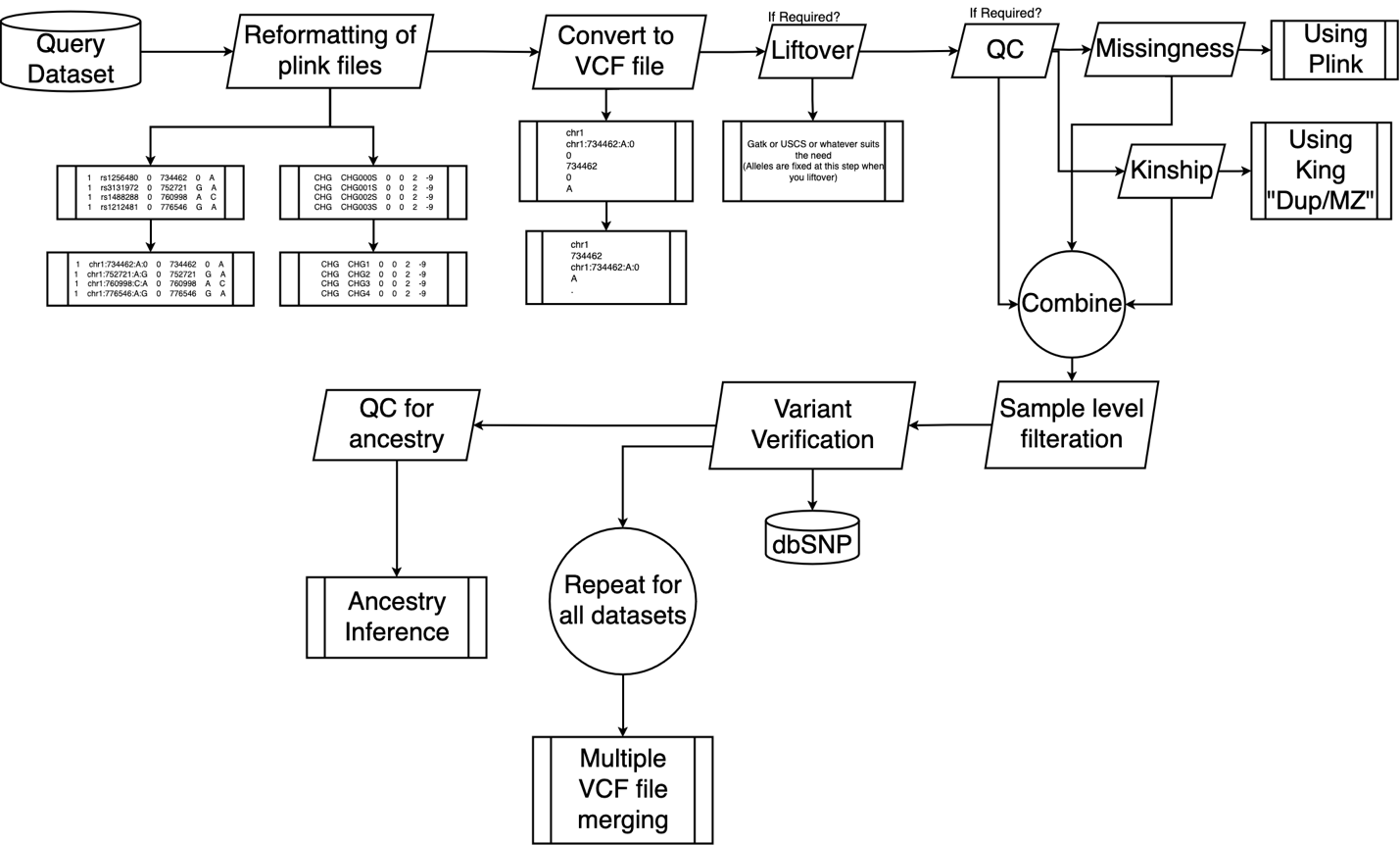


Supplementary Table 1. **CÓDIGO datasets.** The numbers (n) of samples and genomic variants before and after variant merging and harmonization are shown for each source dataset.

| **Source code^a^** | **Source description^b^** | **n samples before** | **n variants before** | **n samples after** | **n variants after** |
| --- | --- | --- | --- | --- | --- |
| CHG | Afro-Colombian from Chocó | 100 | 568,662 | 100 | 567,184 |
| PLQ | Afro-Colombian from San Basilio de Palenque | 34 | 10,064,050 | 34 | 9,779,781 |
| IND | Indigenous Arhuaco, Curripaco, Emberá, Guahibo, Inga, Kogi, Piapoco, Waunana, Wayuu communities | 50 | 364,430 | 50 | 296,141 |
| SIN | Indigenous Sinú community | 19 | 1,407,123 | 19 | 1,459,520 |
| CLM | Mestizo Colombian from Medellín | 94 | 81,568,731 | 94 | 81,568,727 |
| MCM | Mestizo Colombian from Medellín | 524 | 24,181,263 | 373 | 23,188,190 |
| MCA | Mestizo Colombian from Antioquia | 624 | 541,134 | 623 | 526,935 |
| VDC | Mestizo Colombian from Valle del Cauca | 116 | 6,481,171 | 116 | 6,481,171 |

^a^ Three letter code for each source dataset

^b^ Ethnicity and geographic origins for each source data set

Supplementary Table 2. **Global reference populations.** African, American, and European reference populations and samples used for genetic ancestry inference. Population descriptions taken from The International Genome Sample Resource (ISGR): <https://www.internationalgenome.org/data-portal/population>. 1KGP = 1000 Genomes Project; HGDP = Human Genome Diversity Project.

| **Name** | **Description** | **Superpopulation** | **n** | **Source** |
| --- | --- | --- | --- | --- |
| Esan | Esan in Nigeria | African | 99 | 1KGP |
| Gambian Mandinka | Gambian in Western Division, The Gambia - Mandinka | African | 106 | 1KGP |
| Yoruba | Yoruba in Ibadan, Nigeria | African | 107 | 1KGP |
| Karitiana | Karitiana in Brazil | American | 12 | HGDP |
| Maya | Maya in Mexico | American | 13 | HGDP |
| Peruvian | Peruvian in Lima, Peru | Admixed American | 14 | 1KGP |
| Pima | Pima in Mexico | American | 12 | HGDP |
| Surui | Surui in Brazil | American | 8 | HGDP |
| British | British in England and Scotland | European | 87 | 1KGP |
| Iberian | Iberian populations in Spain | European | 86 | 1KGP |
| Toscani | Toscani in Italy | European | 100 | 1KGP |

Supplementary Figure 2. **CÓDIGO development stack.** Server-side components used for the CÓDIGO webserver are shown.


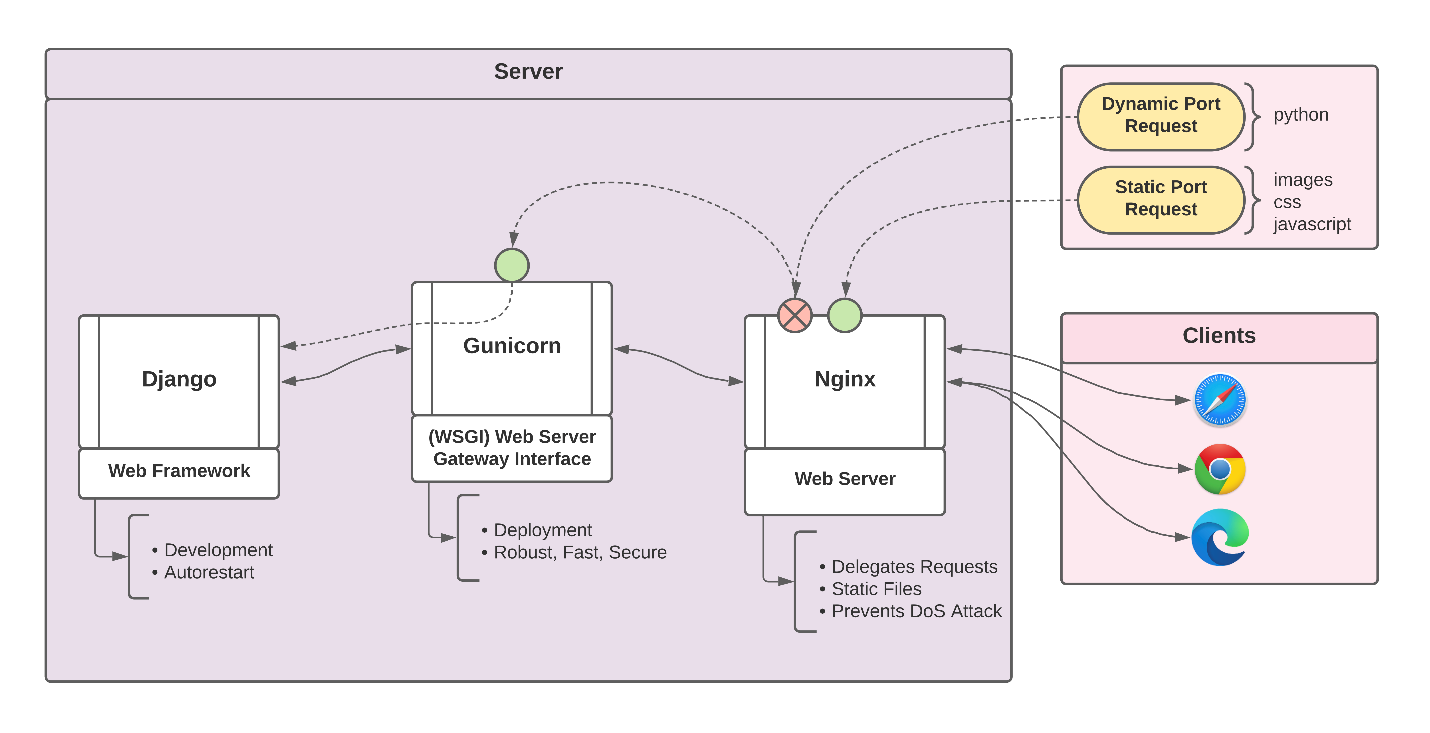


Supplementary Figure 3. **K-means clustering elbow plot.** Sum of squared errors (y-axis) plotted against the number of clusters (k; x-axis) for K-means clustering of CÓDIGO sample genetic ancestry fractions.


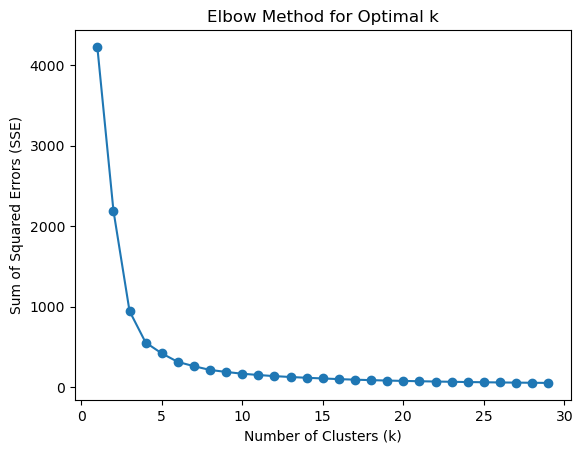

Supplement: Supplement 1 — Supplementary Figure 1. Variant merging and harmonization. Supplementary Figure 2. CÓDIGO development stack. Server-side components used for the CÓDIGO webserver are shown. Supplementary Figure 3. K-means clustering elbow plot. Sum of squared errors (y-axis) plotted against the number of clusters (k; x-axis) for K-means clustering of CÓDIGO sample genetic ancestry fractions. Supplementary Table 1. CÓDIGO datasets. The numbers (n) of samples and genomic variants before and after variant merging and harmonization are shown for each source dataset. Supplementary Table 2. Global reference populations. African, American, and European reference populations and samples used for genetic ancestry inference. Population descriptions taken from The International Genome Sample Resource (ISGR): https://www.internationalgenome.org/data-portal/population. 1KGP = 1000 Genomes Project; HGDP = Human Genome Diversity Project. [file media-1.docx]
